# Experimental and Numerical Investigation of the Die Swell in 3D Printing Processes

**DOI:** 10.3390/mi14020329

**Published:** 2023-01-27

**Authors:** Stefano De Rosa, Daniele Tammaro, Gaetano D’Avino

**Affiliations:** Dipartimento di Ingegneria Chimica, dei Materiali e Della Produzione Industriale, Università Degli Studi di Napoli Federico II, P.le Tecchio 80, 80125 Napoli, Italy

**Keywords:** 3D printing, die swell, strand diameter, numerical simulations, viscoelastic fluids, multi-mode constitutive equations

## Abstract

Fused deposition modelling is one of the most widely used additive manufacturing techniques and the diffusion of 3D printers has increased in popularity even further in recent times. Since high precision is required in 3D printing, a good control over the extrusion process is necessary. In this regard, a crucial phenomenon to be accounted for is the die or extrudate swell, i.e., the enlargement of the cross-section of the strand when coming out of the printer nozzle. While this phenomenon has been studied in large scale extruders, it has not yet been investigated in depth for 3D printing processes. In this work, the die swell phenomenon observed in a printed PLA filament is studied by experiments and fluid dynamic simulations. A novel, easy-to-use, accurate and fast procedure for measuring the value of the die swell ratio during the printing process is developed, accounting for typical errors related to a non-constant strand diameter and possible oscillations of the filament with respect to the extrusion direction. As the printing velocity is increased, a linearly increasing swelling ratio is observed at low printing speeds. The trend flattens at moderate speed values. A sudden increase is found at high printing velocities. The swelling ratio measured with the proposed technique is compared with the results of multi-mode viscoelastic simulations at different temperatures. A fair agreement between the experimental measurements and the numerical predictions is found for printing velocities that are typically employed in commercial 3D printers, supporting the reliability of the developed procedure.

## 1. Introduction

Additive manufacturing (AM) techniques have been employed both at industrial level from medium to high scales, and at lower scales in households, offices, and research centers. As a result of their widespread use in a variety of applications, the market for AM has been rapidly growing in recent years [1,2]. In this context, one of the most widely used techniques is fused deposition modelling (FDM), also known as material extrusion or fused filament fabrication. This technique builds 3D objects starting from a solid polymeric filament that is fed to a heated liquefier, acting both as the feedstock for the extrusion process and as a piston that provides the pressure allowing the material to flow. While the filament is being melted by the heat provided in the liquefier, it is forced through a nozzle and extruded. Three-dimensional printers are machines commonly used in material extrusion in which a motor moves the print nozzle in the three spatial directions as the strand of material is deposited on a flat surface. Three-dimensional objects of many complex shapes are then built by deposition of further layers that solidify on top of each other. For this reason, the filaments are usually made of amorphous or slow crystallising polymers, since fast crystallization would not allow the freshly deposited strand to weld with the previously printed layer [3]. The most commonly used polymer in FDM is polylactic acid (PLA), on which many research works have focused on [4,5].

The reduction in costs and control improvements for extruders and 3D printers have strongly contributed to the increase in popularity of FDM techniques in recent years. The future growth of FDM is, however, still limited by the understanding of the extrusion and deposition processes and the improvement in the manufacturing systems [1,6]. The complexity of the large number of physical phenomena involved requires a strong research effort to improve the performance and optimize the process. In this framework, both experimental and simulation works are useful.

One crucial aspect of controlling the extrusion processes is the understanding of the flow behavior both inside and outside the liquefier, linked to all the properties of the viscoelastic fluids employed in 3D printing that have a nonlinear dependence on shear rate and temperature. Once the filament is melted, the fluid encounters a contraction in the conical part of the nozzle and undergoes both shear and elongational stresses [3]. When the polymer melt is released from the nozzle, it is no longer constrained by the wall and the presence of a free surface allows it to release its stored elastic energy by enlarging its transversal size, giving rise to a phenomenon known as *die swell* or *extrudate swell*. In a Newtonian fluid, the die swell is explained by the rearrangement of the velocity profile, while in viscoelastic fluids, the extrudate swell is mainly attributed to elastic recovery [7,8,9].

A current challenge in FDM processes is the design of the deposition strategy, taking into consideration the polymer extrudate swell that affects the size of deposited strands and the accuracy of the built structure [10,11,12]. When the swell is not considered, the quality of the 3D printed objects is poor [13]. Despite its relevance, the extrudate swell has rarely been investigated in 3D printers. Currently, the design of the deposition strategy relies only on empirical methods and the effect of the processing conditions (e.g., shear rate and temperature) is neglected in standard FDM software. In addition, despite the many investigations that have been carried out on flow defects, the measurements of die swell are often not consistent among different experimental works [14,15,16]. This is due to the absence of a standard procedure for measuring the equilibrium value of the diameter because of a transient zone between the nozzle and the equilibrium of the strand and the existence of oscillations in the strand and in the temperature of the nozzle. It is worth noting that, when increasing the flow rate, three kinds of flow instabilities may occur. First, small defects on the extrudate surface, known as sharkskin, are encountered. Then, at higher flow rates, pressure oscillations cause stronger surface defects. Finally, if the flow rate is further increased, melt fractures occur [17]. Of course, the onset of melt fractures sets an upper limit for swelling measurements.

Several simulations regarding the die swell in extrusion processes have been performed, investigating different aspects of the phenomenon. The first numerical works date back to over 30 years ago and were limited to 2D geometries and simple constitutive equations due to the reduced computational power available at that time [18,19,20]. In recent years, extrudate swell simulations have become increasingly more complex, accounting for accurate viscoelastic and viscoplastic models [21,22], 3D channel geometries [23,24,25], temperature variation, and thixotropy [26]. It is broadly acknowledged that single mode viscoelastic models do not describe accurately enough the non-linear properties of polymeric melts [27]; thus, a quantitative prediction of the extrudate swell phenomenon requires the use of multi-mode viscoelastic constitutive Equations [28,29,30]. Only a few works, however, have compared experimental evidence with simulation results [28,30]. Furthermore, to the authors best knowledge, the analyses of the die swell in commercial 3D printers at small characteristic scales are much more limited.

In this work, we carried out a detailed analysis of the die swell from PLA extrusion footage in a commercial 3D printing device through both experiments and numerical simulations. We developed a novel procedure to accurately measure the diameter of the extruded strand in time at different printing velocities and temperatures, accounting for all the possible issues that might affect the accuracy of the measurements. We have compared the experimental outcomes with fluid dynamic simulation results by employing a multimode viscoelastic constitutive equation with parameters obtained by fitting the PLA rheology.

## 2. Materials and Methods

### 2.1. Experiments

#### 2.1.1. Materials

Polylactic acid (PLA), grade 710M, was supplied by BewiSynbra (Sweden) in the form of pellets. This grade is characterized by a content of the isomer D-PLA equal to ca. 5.5 wt% and a polydispersity index (PI) equal to ca. 3. The PLA pellets were dried overnight at 60 °C in an oven under vacuum conditions before any manipulation. The drying protocol was based on work in the literature data that reported an efficient removal of humidity after 12 h at 60 °C [31]. A PLA filament with a diameter of 1.75 mm was produced using a Composer350 extruder (from 3DEVO company, Utrecht, The Netherlands). The diameter of the filament was measured and controlled by the Composer350 extruder setup.

#### 2.1.2. Experimental Apparatus

**Figure 1:**
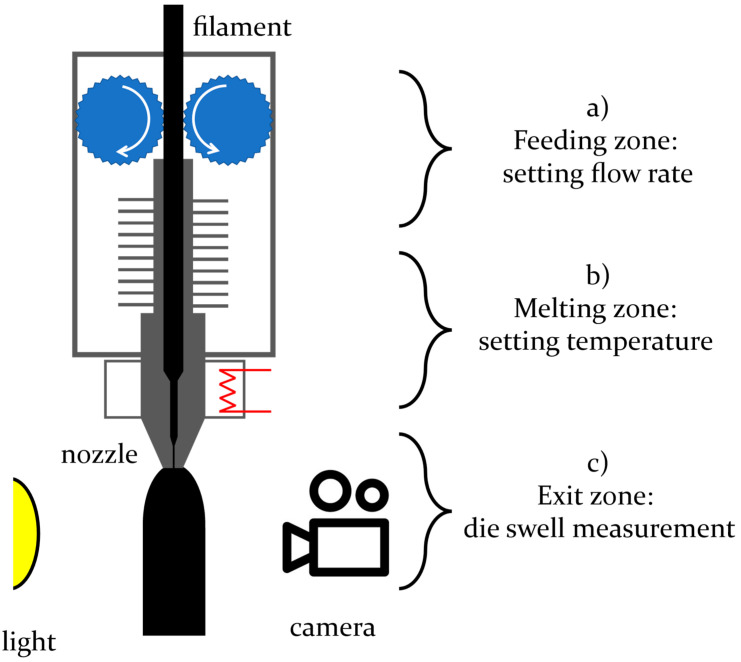
Sketch of the 3D printing setup. (**a**) Feeding zone: setting flow rate; (**b**) Melting zone: setting temperature; (**c**) Exit zone: die swell measurement.

A Prusa Research I3 Mk3s 3D printer was used to extrude the filament, shown in Figure 1. The experimental setup consists of a commercial 3D printer in which three zones can be identified: (a) the feeding zone, where the PLA filament is fed at constant speed by setting the speed of two counter-rotating wheels through a stepper motor (Nema 17) (Stepperonline, New York, NY, USA); (b) the melting zone, where the filament is fused by heat conduction with the metal walls kept at a constant temperature with the action of a 12–140 W cylindrical resistor (RepRap, Wateringen, The Netherlands) and a NPT sensor (RepRap, Wateringen, The Netherlands) with an accuracy of one degree Celsius; and (c) the exit zone, where the extruded strand swells and is recorded by a CCD videocamera (DMX TIS 1/1.8″ CMOS 3072 × 2048 Monocro from DB Electronics, Milan, Italy) equipped with an optical lens (2.0×, 2/3″ SilverTL Telecentric Lens from Edmund, York, UK) aligned at the nozzle exit to record the fluid flow. The use of the 2.0× optical lens allows a resolution of 3 microns/pixel for the calculation of the volumetric flow rate. The background is illuminated by an LED diffused light.

**Figure 2:**
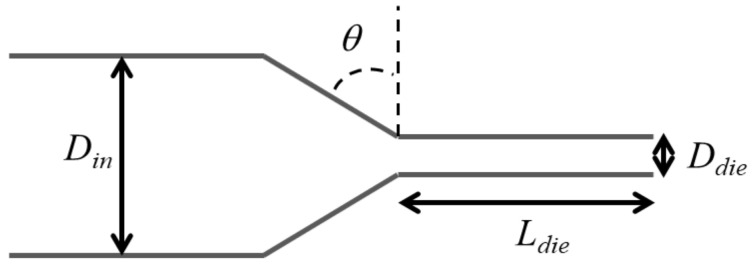
Geometry of the nozzle used for the experiments.

The experiments were performed using a nozzle shaped as shown in Figure 2. The inlet diameter of the nozzle was $D_{\mathrm{in}}=2$ mm, the outlet diameter was $D_{\mathrm{die}}=0.6$ mm, the convergence angle, θ, was 60${}^{{}^{\circ}}$ and the land length, $L_{\mathrm{die}}$, was 1.2 mm. The printer was controlled by using a software called Pronterface (RepRap, USA) that allowed setting of the extrusion speed of the filament (in mm/min), the temperature of the metal nozzle (in °C) and the length to extrude (in mm). The images of the extruded strands at the nozzle exit were acquired by means of the Imaging Source software (The Imaging Source Europe GmbH, Bremen, Germany) at 17.3 frames per second which were saved as movies in AVI format.

#### 2.1.3. Experimental Protocol

The experimental procedure consists of: (a) filament loading, (b) temperature rise and stabilization for at least 10 min after the desired value is reached and (c) extrusion at a constant extrusion speed for 100 mm and, in parallel, optical observation of the die swell through camera recording. We have chosen a design of experiments (DoE) in which the temperature, *T*, was set at three different values above the melting point of PLA, i.e., 160 °C, 180 °C and 200 °C, and the extrusion speed, $u_{\mathrm{in}}$, was varied by choosing 16 values from 5 to 500 mm/min. Both the temperature and extrusion speeds correspond to typical values in 3D printing processes. In total, the presented DoE includes a set of 48 different experiments and a subset of five experiments, randomly chosen, that were repeated to check the consistency of the results.

A consistent measurement of the die swell needs to consider the following aspects: (a) the effect of the gravitational forces on the shape of the strand, (b) the transient zone of the strand diameter from the nozzle exit to the steady zone, (c) the vibrations and oscillations of the exiting strand and (d) the oscillation of the flow rate and temperature due to the 3D printing process. In our experiments, the effect of the gravity was neglected because the viscous forces were higher than the gravitational ones, i.e., the Galilei number, $Ga=gL^{3}/\nu$ (where *g*, *L* and ν are the gravitational acceleration, the characteristic size of the strand and the kinematic viscosity, respectively), is much smaller than one.

**Figure 3:**
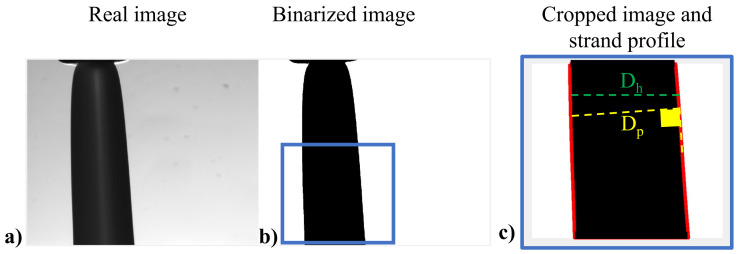
Procedure for die swell measurements. (**a**) Real image showing a snapshot of the recorder movie. (**b**) Binarized image with a box selected by the operator. (**c**) Cropped image from which the horizontal (green dashed line) and perperndicular (yellow dashed line) diameters, $D_{h}$ and $D_{p}$, are calculated.

We have developed a custom-made Matlab script to evaluate whether the strand diameter was constant (after the transient zone) and to provide a consistent measure of this diameter considering both the oscillations of the strand and any possible oscillation in time of the flow rate and temperature. The procedure performs the following operations to analyze the images: (i) It loads the recorded movie of the die swell and extracts all the images, as shown in Figure 3a. (ii) Each image is binarized using the function imbinarize with a constant threshold value equal to 0.4; then, the operator needs to select a rectangle to crop the image in the zone where the diameter does not change qualitatively, as shown in Figure 3b. (iii) The profile of the strand is recognized and stored as two vectors, by means of the function bwboundaries, represented by the red lines in Figure 3c. (iv) Two diameters are measured on the cropped image: the horizontal diameter ($D_{h}$) equal to the horizontal distance between two points with the same ordinate on the recognized red profile (shown in Figure 3c with a dashed green line) and the perpendicular diameter ($D_{p}$), equal to the minimum distance between a point and the other side of the red profile (i.e., the transversal line to the strand axis, shown in Figure 3c with a dashed yellow line). $D_{p}$ is calculated as the product of the $D_{h}$ and the cosine of the angle formed between $D_{h}$ and $D_{p}$. Hence, $D_{p}$ is equal to $D_{h}$ when the strand is perfectly vertical and it is smaller when the strand has a slope with respect to the extrusion direction. By calculating $D_{p}$, it is possible to avoid any error in the diameter measurement due to strand oscillations.

For each image, the script calculated *N* values of $D_{p}$ and $D_{h}$, where *N* is the vertical dimension, in pixels, of the cropped image. In our experiments, *N* ranged from 500 to 1000 depending on the manual cropping. These values were averaged and the standard deviation was calculated. A high standard deviation is either an indication that the surface is very rough (e.g., melt fracture) or that the strand is still in the transient region where the diameter is growing.

#### 2.1.4. Fluid Rheology

**Figure 4:**
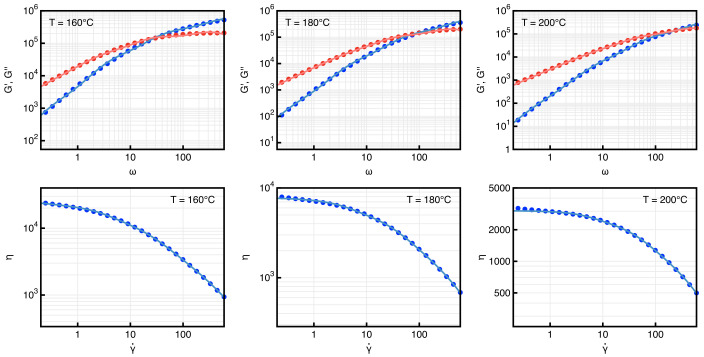
Elastic (red) and viscous (blue) moduli as a function of the frequency (**top** row) and complex viscosity as a funtion of the shear rate (**bottom** row) for the three temperatures. The symbols are experimental measurements and the curves are the predictions of the 4-mode Giesekus constitutive equation.

The viscosity of PLA was measured with a Physica MCR 301 rheometer from Anton Paar, Germany. The measurements were performed under a nitrogen atmosphere at different temperatures (160 °C, 180 °C and 200 °C) using a disposable plate–plate system with a radius of 12.5 mm (PP25) under oscillatory conditions. The gap between the plates was set to 1 mm. The sample was changed for each measurement and at each temperature, and the complex viscosity was determined through a frequency sweep test after 10 min of waiting time to allow the sample temperature to stabilize. The strain amplitude was 10%, and the angular frequency varied from 0.1 to 600 rad/s. The strain amplitude was determined by means of a dynamic strain amplitude sweep in order to be in the linear viscoelastic regime. The linear viscoelastic moduli, $G^{\prime }$ and $G^{\prime \prime }$, and the complex viscosity, η, are shown as symbols in Figure 4 for the three temperatures.

### 2.2. Numerical Simulations

#### 2.2.1. Mathematical Model

**Figure 5:**
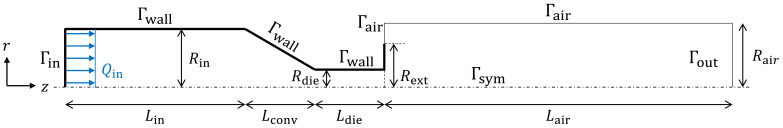
Schematic representation of the simulation domain.

Since the nozzle geometry has an axis of symmetry, the problem was simulated in a 2D axisymmetric domain, as shown in Figure 5. The thicker lines represent the boundaries of the nozzle, whereas the thinner ones are the boundaries of the external domain.

The dimensions of the domain were chosen to match the nozzle of the 3D printer and are reported in Table 1. The values of $L_{\mathrm{in}}$, $L_{\mathrm{air}}$ and $R_{\mathrm{air}}$ were chosen to be large enough to neglect any effect of the inlet and outlet boundary conditions on the simulation results. The origin of the axes was placed at the exit of the nozzle on the symmetry axis.

The simulations were carried out by solving the mass and momentum balance equations governing the fluid dynamics of the melted polymer through the nozzle and in the surrounding environment (air). Hence, a multiphase system (PLA + air) was considered. We assume incompressible fluid and laminar flow conditions, justified by the very low values of the Reynolds numbers (see later). Gravity effects were negligible as discussed above. Even though temperature variations were present in the problem, for the sake of simplicity, the fluid was assumed to be at the temperature imposed by the heated walls of the liquefier, thus the problem was considered isothermal. This is a reasonable assumption at relatively low flow rates, as the temperature in the liquefier has time to become homogeneous.

Under these assumptions, the mass and momentum balance equations for the PLA read as:

(1) ∇·u=0

(2) $$\rho \left(\frac{\partial u}{\partial t}+u\cdot \nabla u\right)=-\nabla p+\nabla \cdot \sigma$$

where u, *p*, ρ and σ are the fluid velocity, pressure, density and the viscoelastic stress tensor, respectively. The latter is expressed through sum of *M* modes:

(3) $$\sigma =\sum_{n=1}^{M}\sigma_{i}$$

where the stress tensor of each mode is given by the Giesekus constitutive Equation [32,33]:

(4) $$\lambda_{i}\overset{\nabla }{\sigma_{i}}+\sigma_{i}+\frac{\alpha_{i}\lambda_{i}}{\eta_{i}}\sigma_{i}\cdot \sigma_{i}=2\eta_{i}D$$

In this equation, $\lambda_{i}$, $\alpha_{i}$ and $\eta_{i}$ are the relaxation time, the mobility and the polymer viscosity of the *i*-th mode, respectively, D is the rate-of-deformation tensor:

(5) $$D=\frac{1}{2}\left(\nabla u+{\left(\nabla u\right)}^{T}\right)$$

and $\overset{\nabla }{\sigma_{i}}$ is the upper-convected time derivative that assures frame invariance:

(6) $$\overset{\nabla }{\sigma_{i}}=\frac{\partial \sigma_{i}}{\partial t}+u\cdot \nabla \sigma_{i}-{\left(\nabla u\right)}^{T}\cdot \sigma_{i}-\sigma_{i}\cdot \nabla u$$

The use of a multi-mode Giesekus model to describe viscoelastic fluids is well documented in the literature for various flow problems [34,35,36].

The number of modes, *M*, and the constitutive parameters were obtained by fitting the model predictions with the rheological data presented above. Specifically, a regression of the linear viscoelastic moduli gives *M*, the relaxation times and the polymer viscosities. The mobilities were then estimated by fitting the complex viscosity data. We found that four modes were sufficient to accurately describe the PLA rheology. The model predictions are shown as solid curves in Figure 4. The values of the constitutive parameters at the three temperatures are listed in Table 2.

The equations governing the fluid dynamics of the air are the same as Equations (1) and (2) with $\sigma =2\eta_{\mathrm{air}}D$ and ρ replaced by the air density, $\rho_{\mathrm{air}}$. The surface tension force, γ, acts at the liquid–air interface. The other physical parameters used in the simulations are listed in Table 3.

#### 2.2.2. Boundary and Initial Conditions

With reference to Figure 5, a fixed velocity profile is set in inlet, $\Gamma_{\mathrm{in}}$, with a constant velocity, $u_{\mathrm{in}}$, in the axial direction *z*, as imposed from the printer control software. On the outlet boundary, $\Gamma_{\mathrm{out}}$, and on the boundaries of the air domain, $\Gamma_{\mathrm{air}}$, a constant pressure equal to the ambient one is applied. Due to the fluid incompressibility, we can set such a pressure level equal to 0. At the nozzle walls, $\Gamma_{\mathrm{wall}}$, no-slip conditions are imposed, whereas on the symmetry axis, $\Gamma_{\mathrm{sym}}$, a symmetry condition is set. Finally, a boundary condition for the extra stress tensor, σ, is required in inflow. We set σ=0. The choice of $L_{\mathrm{in}}$ assures that the velocity and stress fields fully develop in the inlet channel before entering the contraction.

**Figure 6:**
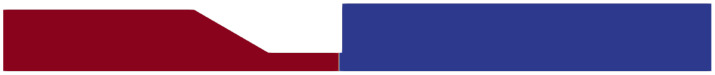
Initial conditions for the simulations carried out in this work. The domains occupied by the PLA and air are in red and blue, respectively.

The problem was initialized with velocity, pressure and stress identically null in the whole domain. In the initial conditions, the PLA occupied the whole nozzle domain up to 0.05 mm from the the end of the die, whereas air fills the rest of the domain, as shown in Figure 6, in which PLA is represented in red and air in blue.

#### 2.2.3. Dimensionless Numbers

From the model parameters in Table 2, we can compute the relevant dimensionless numbers for the problem; namely, the Reynolds, Weissenberg and capillary numbers:

(7) $$Re=\frac{\rho \;u_{\mathrm{in}}\;D_{\mathrm{in}}}{\eta_{0}}\;Wi=\frac{\lambda \;u_{\mathrm{in}}}{D_{\mathrm{in}}}\;Ca=\frac{\eta_{0}\;u_{\mathrm{in}}}{\gamma }$$

The characteristic length was chosen as the inlet diameter $D_{\mathrm{in}}=2$ mm, while the characteristic velocity was the average inlet velocity, $u_{\mathrm{in}}$. The maximum relaxation time was selected to define the Weissenberg number and the zero-shear viscosity, $\eta_{0}$, was used in the definition of the Reynolds number. Table 4 reports the values of Re, Wi and Ca for the maximum average velocity considered in this work and for the three investigated temperatures. It can be readily observed that the Reynolds number was very low, as typically occurs for these systems. A Weissenberg number of up to about eight was obtained, denoting the relevance of elastic effects. The capillary number was quite high, meaning that the viscous forces are much stronger than the surface tension ones.

#### 2.2.4. Simulation Software

All the simulations have been performed using RheoTool (version 5.0) [37,38], which is a toolbox of the open-source software OpenFOAM, specifically designed to deal with viscoelastic fluids. The solver used is *rheoInterFoam,* a transient solver for two-phase laminar, isothermal flows that combines the *interFoam* solver of OpenFOAM with the *rheoFoam* solver of RheoTool to simulate multiphase viscoelastic flows. This solver is based on the volume-of-fluid (VOF) approach to capture the interface between the two phases. In this method, the mass and momentum balance equations of the two fluids are combined in a single set of equations. A new variable, termed ‘indicator function’ or ‘color function’, denoted by α, is added to identify the regions of the domain occupied by one of the two phases. Specifically, the PLA and air domains are identified by α=1 and α=0, respectively. The liquid–air interface corresponds to α=0.5. This variable is transported by the fluid velocity so a convection equation for the indicator function is added to the mathematical model. The physical properties in the combined Equations (e.g., density and viscosity) are weighted between the properties of the single phases through the indicator function. The indicator function requires initial and boundary conditions. The initial condition is set as shown in Figure 6, i.e., we set α=1 in all the mesh cells occupied by PLA (red) and α=0 elsewhere (blue). The boundary conditions are α=1 on $\Gamma_{\mathrm{in}}$, since only the viscoelastic fluid enters the domain, a symmetry condition on $\Gamma_{\mathrm{sym}}$ and a zero-gradient condition on the other boundaries.

The software solves the transient problem described by the combined mass and momentum balance equations, the viscoelastic constitutive equations, and the convective equation for the indicator function. We set a sufficiently large final time to let the velocity and stress fields as well as the extrudate profile attain a steady-state. The die swell ratio is then computed as the ratio between the diameter of the extrudate in the zone where it has an horizontal profile and the nozzle diameter $D_{\mathrm{die}}$.

#### 2.2.5. Mesh and Mesh Convergence Study

The domain was discretized in quadrilateral elements. A mesh convergence study was performed on a test case at T=180 °C and $u_{\mathrm{in}}=100$ mm/min using four different mesh resolutions that have the same element distribution but a different level of refinement. The details of the meshes used are reported in Table 5.

**Figure 7:**
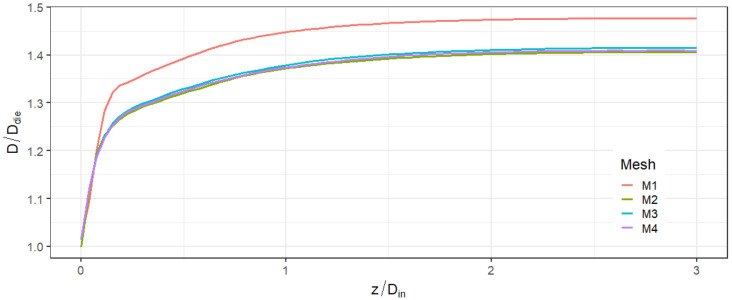
Profile of the extrudate for the four different meshes. The *z*-coordinate is normalized by the inlet diameter, $D_{\mathrm{in}}$. The origin corresponds to the end point of the nozzle.

**Figure 8:**
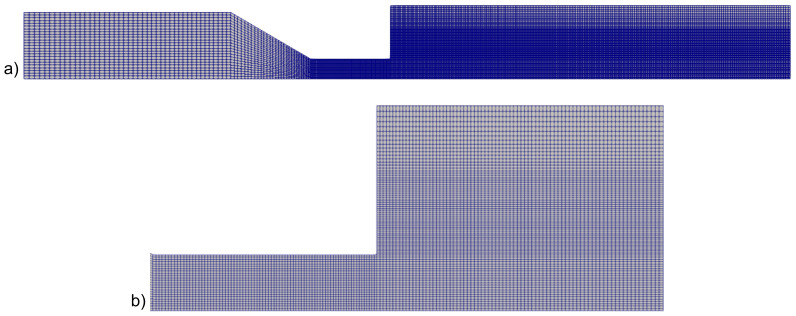
(**a**) Mesh M3 used in the simulations. (**b**) Zoomed-in image of the die and the exit zone (bottom).

In Figure 7, the PLA–air interface outside the nozzle (obtained as the contour corresponding to α=0.5) is plotted against the *z*-coordinate for the four different meshes. Apart from the mesh M1 which is extremely coarse, the extrudate profiles corresponding to the finer meshes are fairly superimposed. By increasing the inlet velocity, we found that the mesh M3 always produces results equivalent with mesh M4. The former was then used in the present work. The complete mesh is shown in Figure 8a together with a zoomed-in image of the die and of the exit zone in Figure 8b.

## 3. Results and Discussion

### 3.1. Experimental Results

**Figure 9:**
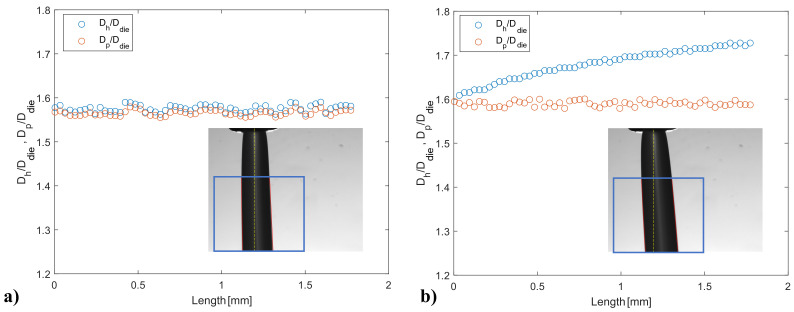
Horizontal and perpendicular strand diameters, $D_{h}$ and $D_{p}$, measured from the custom-made script, as function of the length of the cropped image: (**a**) straight strand and (**b**) bent strand. The two images are taken at different instants of the same experiment at 160 °C and 100 mm/min.

Figure 9 shows the horizontal and perpendicular diameters of the strand, $D_{h}$ and $D_{p}$, as function of the vertical length of the cropped image as obtained by the custom-made script. The origin of the abscissas in Figure 9 is set at the top horizontal boundary of the cropped image (blue box). Hence, the values on the *x*-axis of the graphs correspond to the vertical distance from the top boundary of the cropped image. Both diameters are normalized by the die diameter $D_{\mathrm{die}}=2R_{\mathrm{die}}$. Panels (a) and (b) refer to two images taken at different instants of the same experiment, i.e., 160 °C and 100 mm/min. In Figure 9a, the two sets of data are very close over the entire length of the cropped image. Indeed, the strand shown in the inset is almost vertical and its borders (denoted by red lines) are almost parallel to the yellow dashed axis that represents the extrusion direction. Moreover, the data in the graph are horizontal, meaning that the strand diameter is constant in the cropped region. On the contrary, the blue and red dots in Figure 9b are close at small lengths but deviate with increasing the length. In this case, the strand shown in the inset is tilted with respect to the extrusion direction. The increase of $D_{h}$ is due to the slope of the strand and not to a real increase in the diameter. The perpendicular diameter is, in fact, the most accurate measurement of the die swell because it takes under consideration the slope of the strand and its oscillations during measurement.

**Figure 10:**
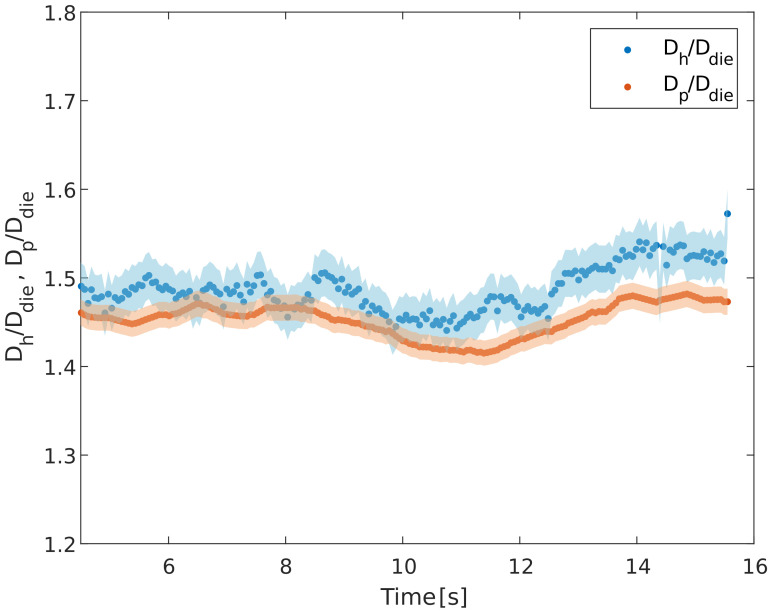
Time evolution of the normalized horizontal (blue) and perpendicular (red) diameters for an experiment at 160 °C and 100 mm/min. The bands around the data represent the standard deviation.

Figure 10 reports the results of the die swell as a function of the time for the experiment performed at 160 °C and 100 mm/min. Each point in Figure 10 is the average of the horizontal and perpendicular diameters over the length of the cropped image (as shown in Figure 9) and the shaded areas are the standard deviations. Notice that $D_{h}$ (blue dots) has a bigger error and higher fluctuation frequency with respect to $D_{p}$ (red dots) due to the aforementioned vertical oscillations of the strands during the experiments. However, the measurements show some fluctuations in $D_{p}$ as well. These can be attributed to the temperature fluctuation (with a period of around 10 s) in the process, related to the PID control of the temperature, as also reported in the literature [4]. The consistency of the measurements was checked by repeating the experiments three times at five different velocities for the lowest and the highest temperature. The results fall within the experimental error, shown in Figure 10.

In the rest of the work, the experimental results will be reported only in terms of $D_{p}$ as, as previously mentioned, it is a more accurate estimation of the real strand diameter.

### 3.2. Simulations Results

Simulations were performed with different values of inlet velocity ranging from 20 to 400 mm/min. The effect of the temperature and inlet velocity on the fluid rheological properties was evaluated by comparing, in dimensionless form, the relevant components of the velocity and stress tensor in the converging zone of the nozzle, in the die and in the exit zone of the fluid for three sets of *T* and $u_{\mathrm{in}}$.

**Figure 11:**
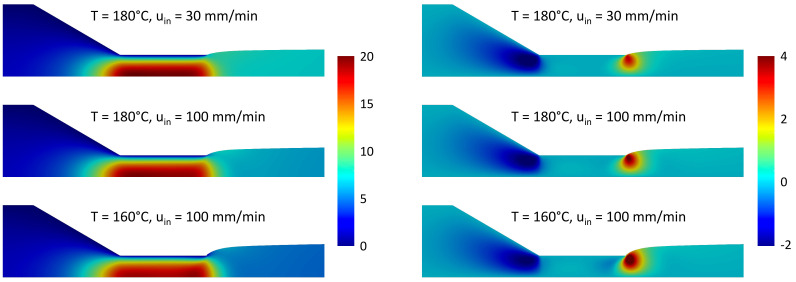
Axial (**left**) and radial (**right**) components of the polymer velocity for three sets of simulation parameters.

As discussed in Section 2.2.3, the velocity components were made dimensionless by the inlet velocity and the stress components by $u_{\mathrm{in}}\eta_{0}/D_{\mathrm{in}}$. In Figure 11, the dimensionless axial and radial components, $u_{z}$ and $u_{r}$, of the velocity are shown. The differences inside the inlet channel and the first part of the die are minimal in all three simulations and for both axial and radial components, as expected for a fully developed flow. In the exit zone of the die and in the first part of the extruded strand, there are quantitative differences in the velocity. When the inlet velocity increases or the temperature decreases, the swelling of the viscoelastic fluid increases and, as a consequence, the radial component of the velocity increases in proximity of the channel exit. The axial component, instead, decreases in magnitude with the increase in the swelling ratio, and hence of the transversal area, due to the mass conservation. In all the simulations, the velocity profile becomes a plug-flow far from the die exit, as also reported in the literature [39].

**Figure 12:**
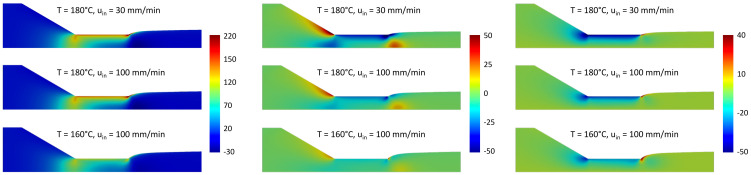
zz (**left**), rr (**middle**) and rz-component (**right**) of the stress tensor for three set of simulation parameters.

In Figure 12, the relevant components of the stress tensor, $\sigma_{\mathrm{zz}}$, $\sigma_{\mathrm{rr}}$ and $\sigma_{\mathrm{rz}}$. are plotted in dimensionless form for the same three simulations. For all three components, as the temperature decreases or the velocity increases, the stress increases both in the nozzle contraction and at the exit of the die. A large stress region is, in particular, observed at the contact point between the polymer and air at the end of the die due to the geometric singularity.

### 3.3. Die Swell Ratio: Experiments vs. Simulations

**Figure 13:**
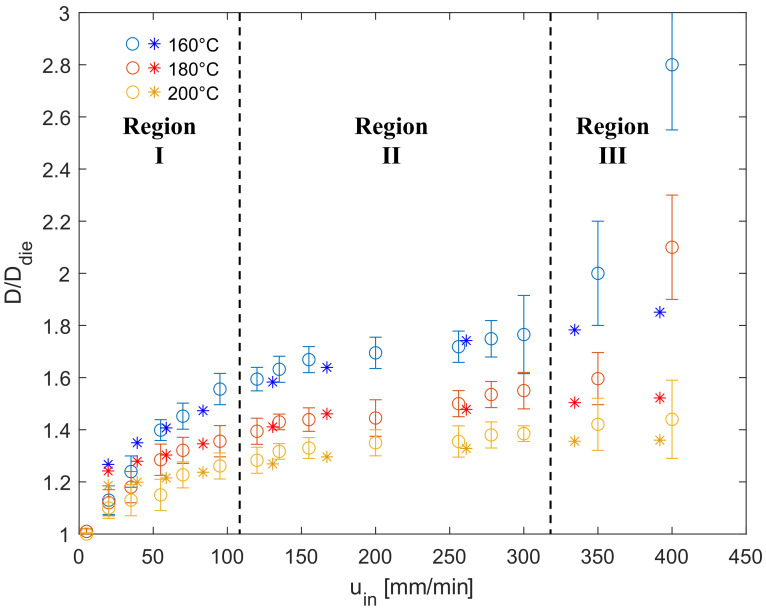
Die swell as function of velocity at three temperatures. Comparison between experimental (circles) and simulation results (stars).

Figure 13 shows the die swell ratio, $D/D_{\mathrm{die}}$, at the three temperatures as a function of the printing speed. The circles are the experimental measurements and the stars denote the simulation results. For all the investigated temperatures, the die swell ratio increases monotonically as a function of the printing speed, while it decreases by increasing the temperature from 160 °C to 200 °C due to the reduction in the normal stresses. By looking at the experimental measurements, the data show an inflection point at a velocity of about 200 mm/min. In particular, three regions can be identified: (i) Region I, from 0 to about 100 mm/min (with a range that slightly depends on the temperature), is characterized by an almost linear increase in the die swell ratio with a slope that decreases with increasing temperature. (ii) Region II, from 100 to about 350 mm/min, is characterized by a very weak increase in the die swell ratio with the printing velocity. (iii) Region III, from 350 to 500 mm/min, is characterized by a sudden increase in the die swell ratio. Notice that, in this latter region, the error bars are much larger than those obtained at lower velocities.

In the first two regions, a remarkable agreement between the experiments and the simulations is found. Specifically, the simulations were able to capture both the nearly linear increase in the swelling ratio observed in Region I as well as the flatter trend noticed in Region II. The agreement is also quantitative as most of the simulation points fall within the experimental error bars for all the investigated temperatures. On the other hand, the abrupt increasing trend measured in the experiments at higher velocities (Region III) is not captured by the simulations, as they predict a nearly constant die swell ratio, similar to that in Region II.

A possible justification of the observed results is that at higher printing velocities, the residence time of the filament in the liquefier is not sufficient to reach a homogeneous constant temperature in the entire cross-section of the strand (i.e., the residence time is lower than the heating time). Thus, the sudden increase in the die swell slope in Region III observed in the experiments is likely due to the strong temperature inhomogeneity within the filament, as also reported in the literature [40,41,42], making, in fact, the heat transfer a limiting factor in polymer extrusion, especially at high flow rates. Such a temperature inhomogeneity was not considered in the simulations. Furthermore, even if in the investigated range of printing velocity no melt fracture phenomena were observed, the minor flow instabilities that occur are not easily captured with numerical simulations. It is hence evident how the simulations are able to describe the printing process at low and moderate extrusion velocities; whereas, at higher flow rates, the isothermal assumption is no longer suitable to accurately describe the process. Finally, it has to be mentioned that the die swell phenomenon depends on the shear as well as extensional stresses to which the fluid is subjected inside the nozzle [7]. The calibration of the constitutive parameters was, however, only based on shear viscosity data which is extensional data that is not available and much more difficult to acquire. It is not guaranteed, however, that a good prediction of the shear rheology leads to an accurate description of the extensional stresses too. It might also be possible that the multi-mode Giesekus model employed in this work could not accurately describe the extensional rheology of the PLA. Since at high printing speeds the extensional stresses become more and more important, inaccuracies in the extensional properties would lead to deviations between simulation results and experimental measurements.

## 4. Conclusions

In this paper, we have investigated with experiments and fluid dynamic simulations the die swell phenomenon observed in a 3D printing process. We have developed an automatic technique to measure the strand diameter in both time and space encoded in a easy-to-use script. Such a technique accounts for possible errors that affect the measurement accuracy due to non-constant strand diameters (induced, for instance, by transient effects) and possible oscillations of the strand with respect to the extrusion direction. A filament of PLA has been extruded at several velocities and three temperatures (with a DoE that includes 48 experiments). We have compared the experimental measurements with numerical results obtained by solving the fluid dynamics equations of the polymer flowing in the die and exiting to the external environment. We have employed an open-source code to simulate the dynamics of the multiphase system, with the PLA shear rheology described by a multi-mode Giesekus constitutive equation.

The experimental analysis method turned out to be accurate and useful to quickly control the extent of the swelling at different velocities and temperatures, opening up the possibility to implement the developed script in an in-line control loop. The simulations describe the die swelling ratio for low and moderate printing velocities well at all three investigated temperatures. At high velocities, however, the experimentally measured swelling ratio suddenly increases and the simulations are not able to capture such a phenomenon. This is likely due to thermal effects that give rise to important temperature inhomogeneities within the filament, not accounted for in the modeling. Furthermore, a better description of the extensional rheological properties might be required, possibly selecting more accurate constitutive equations to describe the PLA rheology. The measurement technique and the numerical method employed in this work can be used to investigate the effect of other relevant parameters on the die swelling phenomenon in 3D printing processes, such as the nozzle geometry and use of different polymers. This will be part of future work.

## Figures and Tables

**Table 1:** Dimensions (in mm) of the geometrical parameters used in the simulations.

$L_{\mathrm{in}}$	3.1	$R_{\mathrm{in}}$	1
$L_{\mathrm{conv}}$	1.2	$R_{\mathrm{die}}$	0.3
$L_{\mathrm{die}}$	1.2	$R_{\mathrm{air}}$	1.1
$L_{\mathrm{air}}$	6	$R_{\mathrm{ext}}$	0.75

**Table 2:** Values of the constitutive parameters for the 4-mode Giesekus model at the three temperatures.

T = 160 °C	$\lambda_{i}$ (s)	$\alpha_{i}$	$\eta_{i}$ (Pa · s)
Mode 1	0.264	0.58	11,556
Mode 2	3.13	0.44	5244.4
Mode 3	0.0308	0.51	6943.7
Mode 4	0.002823	0.50	1122.9
**T = 180 °C**	$\lambda_{i}$ **(s)**	$\alpha_{i}$	$\eta_{i}$**(Pa**·**s)**
Mode 1	0.0225	0.50	3124
Mode 2	0.191	0.67	2959
Mode 3	2.407	0.14	791.4
Mode 4	0.0023	0.50	860.2
**T = 200 °C**	$\lambda_{i}$ **(s)**	$\alpha_{i}$	$\eta_{i}$**(Pa**·**s)**
Mode 1	0.0156	0.52	1394
Mode 2	0.126	0.68	907.6
Mode 3	1.547	0.10	149.8
Mode 4	0.00173	0.50	578.2

**Table 3:** Physical parameters for PLA and air.

ρ	γ	$\rho_{\mathrm{air}}$	$\eta_{\mathrm{air}}$
(kg/m ${}^{3}$)	(mN/m)	(kg/m ${}^{3}$)	(Pa·s)
1000	42	1.225	1.8$\cdot 10^{-5}$

**Table 4:** Dimensionless numbers for the three temperatures considering the maximum inlet average velocity investigated in this work.

Temperature	Re	Wi	Ca
160 °C	4.01$\cdot 10^{-7}$	7.80	2.96$\cdot 10^{3}$
180 °C	1.29$\cdot 10^{-6}$	6.02	9.21$\cdot 10^{3}$
200 °C	3.30$\cdot 10^{-6}$	3.87	3.61$\cdot 10^{3}$

**Table 5:** Details of the meshes used in the spatial convergence study.

Mesh	# of Cells	# of Elements on $R_{\mathrm{die}}$
M1	1428	6
M2	5712	12
M3	22,848	24
M4	35,700	30

## Data Availability

Not applicable.
